# Changes in the higher-level functional capacities for modern daily living in community-dwelling stroke survivors: A preliminary case series

**DOI:** 10.3389/fneur.2022.948494

**Published:** 2022-10-19

**Authors:** Tsubasa Kawasaki, Masahiro Ohira, Ryu Endo, Keita Muto, Hiroki Sakai, Keisuke Goto

**Affiliations:** ^1^Department of Physical Therapy, School of Health Sciences, Tokyo International University, Kawagoe, Saitama, Japan; ^2^Department of Rehabilitation, Faculty of Health Sciences, Uekusa Gakuen University, Chiba, Japan; ^3^Department of Rehabilitation, Yokohama Shin-midori General Hospital, Yokohama, Kanagawa, Japan; ^4^Department of Rehabilitation, Tokyo Women's Medical University Adachi Medical Center, Tokyo, Japan

**Keywords:** capacities for modern daily living, instrument activities of daily living (IADL), stroke, community-dwelling, longitudinal case series, activities of daily living (ADL)

## Abstract

Along with the development of modern technology, the variety of higher-level activities of daily living has steadily expanded. However, no reports have examined the capacities for the higher-level activities in stroke survivors. Moreover, the relationship between these capacities and executive functions has not been reported. This preliminary study longitudinally measured changes in the capacities for high-level activities of modern daily living in community-dwelling stroke survivors. In addition, we examined whether changes in these capacities were correlated with executive functions. The results showed no significant reduction in the capacities for high-level activities of modern daily living at 1 year after stroke. Significant improvements were observed in one aspect of executive functions: planning. The changes in the capacities for higher-level activities of modern daily living were significantly correlated with executive function. The results suggest that, in stroke survivors, (a) it is likely feasible to maintain the capacities for higher-level activities of modern daily living, and (b) these capacities are related to executive functions.

## Introduction

Stroke sequelae are serious conditions that cause long-term disruptions to daily life. According to The Global Burden of Disease, Injuries and Risk Factors 2016 study, disease-adjusted life years (DALYs; one DALY is one lost year of full health) are highest for people with neurological disorders; indeed, DALYs from neurological disorders have been increasing rapidly (1). Strokes account for over 42.2% of DALYs, far more than other diseases, such as migraine (16.3%) or Alzheimer disease (AD) and other dementias (10.4%) (1). In recent years, mortality from stroke has decreased because of a reduction in fatal hemorrhages based on the development of approaches to control hypertension (2). However, two-thirds of stroke survivors are forced to live with disabilities. It is assumed that most of the DALYs from stroke are years lost due to disability (3).

Many stroke patients receive rehabilitation intervention from professionals (e.g., physiotherapists or occupational therapists) after discharge from the hospital. This is based on ample evidence of the effectiveness of home-based rehabilitation to improve physical function (4) and capacities for daily living (5, 6). In fact, from the perspective of healthcare cost efficiency, home-based rehabilitation is a common intervention after discharge and is recommended widely (7).

Maintenance of capacities to perform activities of daily living (ADLs) is fundamental to prevent the decline of a stroke survivor's quality of life. ADLs can be divided into basic ADLs (BADLs) and instrumental ADLs (IADLs). BADLs consist of self-care (feeding, bathing, toileting, dressing, and bladder and bowel management) and motility (transfers, ambulation, stair climbing). BADLs are an essential and fundamental set of activities for daily living. On the other hand, IADLs consist of activities using instruments (meal preparation, community mobility, health maintenance, home management (clothing care, cleaning), shopping, and care of others). IADLs are regarded as a set of more applied activities compared with BADLs.

However, along with the spread of modern technology, the set of IADLs has grown to include new activities. The Internet has come to define the modern lifestyle by providing a huge range of information to its users (8). Indeed, the selection and consumption of information from the Internet is now integral to modern daily life. In conjunction with these changes, the concept of social frailty has been proposed in recent years, leading to a growing interest in social participation (9–11). Underlying this growing interest is the understanding that social participation improves cognitive function (12, 13), physical function (14, 15), and quality of life by enhancing interpersonal communication and physical activity (16, 17). These healthful effects of social participation indicate that the framework of existing IADLs should be extended to accommodate the social aspects of modern life. Concomitantly, the content of the assessment of modern IADL capacities should also change according to modern lifestyles.

The Lawton IADL scale is a traditional assessment battery of IADLs and is used widely around the world. However, this scale does not include recent lifestyle activities related to information gathering and social participation. Recently, the JST-IC (Japan Science and Technology Agency Index of Competence) was devised as an index to measure higher-level functional capacities for modern daily living, including items of information practice and social participation (18, 19). Previous studies showed that capacities for daily living were related to activity diversity in community-dwelling older adults (20), going-out frequency (21), physical fitness, subjective health, and social engagement (19). No reports have evaluated community-dwelling stroke survivors using the JST-IC.

In addition, IADL capacities and modern daily living capacities are likely to be related to executive functions, but these relations have not been fully understood. Executive functions are brain functions dedicated to sustaining a proper mental set to accomplish a future goal (22) and consist of inhibitory control, working memory, and cognitive flexibility, which together foster success in all aspects of life (23). IADLs and activities of modern daily living are more complicated than BADLs and require more complex interactions with the environment (24). Executive functions may be involved in the higher-level activities of daily living, because these activities require that an individual plan and efficiently execute various procedures while simultaneously monitoring himself or herself. Indeed, IADL abilities in stroke survivors as measured by the Lawton IADL scale have been shown to be related to executive functions (25). To date, however, the relationships between the capacities for higher-level activities of modern daily living and executive functions have not been elucidated.

The first purpose of this study was to identify changes in the capacities for higher-level activities of modern daily living in community-dwelling stroke survivors. The second purpose was to determine whether changes in the capacities for higher-level activities of modern daily living are related to executive functions. In this report, we present a longitudinal case series examining (a) changes in the capacities for activities of modern daily living and (b) the relationships between the capacities for higher-level activities of modern daily living and executive functions in community-dwelling stroke survivors.

## Methods

### Participants

Eleven chronic stroke survivors in a community-dwelling environment participated (3 men and 8 women; age ± SD: 64.5 ± 10.1 years). The participants were recruited from among patients who had been discharged from Yokohama Shin-midori General Hospital. Each participant received therapy from 1 of 5 randomly assigned visiting rehabilitation therapists, among whom were 3 of the present authors (Ohira, Endo, and Muto). The inclusion criteria were as follows: more than 6 months after stroke onset, a score of more than 80 points on the Functional Independence Measure (FIM) (26), and a score of < 4 points on the Six-Item Screener (SIS) (27, 28). The FIM reflects functional capacities of basic daily living, comprising 18 items in total. Each item is scored on a scale of 1–7, so that the range of total possible scores is 18 to 126. A score of more than 80 points on the FIM indicates independence in ADL (29, 30) or a successful outcome (31). The SIS is a brief and reliable tool for screening participants' cognitive impairment using six items from the Mini-Mental State Examination (i.e., orientation to time and recall). Four points or more on the SIS indicates normal cognitive function (27). The exclusion criteria were moderately or substantially higher brain dysfunction (aphasia, apraxia, or visuospatial deficit) or depression. The presence of aphasia and agnosia was determined using stroke impairment assessment set items 19 and 20 (32). A speech therapist further screened for aphasia by confirming voluntary speech, auditory understanding, tool naming, word repeating, reading, and writing. The presence of apraxia was determined by verbal instruction and imitation of object use. Depression was defined as a score of ≥16 points on a self-rating questionnaire for depression (33).

All participants were provided with comprehensive assessment-based rehabilitation once a week. The rehabilitation consisted of joint range-of-motion exercises, muscle strengthening exercises, stretching, balance exercises, gait exercises, and practice in performing activities of daily living. Table 1 shows the basic characteristics of the participants, duration from stroke onset, hemiplegia side, hemiplegia degree (Brunnstrom stage), and the ability to walk (Timed Up-and-Go test) (34). These tests revealed that no participants showed aphasia or a visuospatial deficit. Slight apraxia was found in participants D, H, and L; however, all participants were able to fully communicate with the examiner and had full BADL capacities (see FIM points in Table 1).

**Table 1:** Basic and clinical characteristics of the study participants at the time of initial assessments (*n* = 11).

**ID**	**Sex**	**Age**	**Duration from onset (months)**	**Injured brain area**	**Hemiplegia side**	**FIM (points)**	**BRS (UE)**	**BRS (Finger)**	**BRS (LE)**	**TUG (sec)**
A	W	80	11	Left corona radiata	R	123	5	5	5	9.8
B	W	54	30	Left frontal lobe	R	115	2	2	3	18.3
C	W	56	25	Left thalamus	L	118	5	6	6	12.5
D	W	72	59	Left frontal lobe	R	123	6	6	6	7.2
E	W	75	12	Left frontal lobe	L	86	5	5	5	30.3
F	W	72	13	Right putamen	L	120	5	5	5	18.5
G	W	47	17	Wide area of the right middle brain artery	L	121	2	2	5	8.6
H	M	66	33	Right putamen	L	122	6	6	6	9.5
I	M	65	10	Right posterior large cerebral artery	L	116	5	5	5	28.1
J	M	65	26	Left putamen	R	117	3	2	4	26
K	W	57	12	Right putamen	R	124	2	2	4	20.4
Mean ± SD	N/A	64.5 ± 10.1	22.5 ± 14.7	N/A	N/A	116.8 ± 10.7	–	–	–	17.2 ± 8.3
Median (25, 75%ile)	N/A	–	–	N/A	N/A	–	5 (2.5.5)	5 (2,5.5)	5 (4.5,5.5)	–

FIM, Functional independence measure; BRS, Brunnstrom recovery stage; UE, Upper extremity; LE, Lower extremity; TUG, Timed up-and-go test.

Participants were given a full oral explanation of the study purposes and methods, and all participants gave written informed consent prior to the start of the measurements. The Institutional Ethics Committee of Yokohama Shin-midori General Hospital approved all experimental protocols (approval number 18011). The tenets of the Declaration of Helsinki were followed.

### Measurements of capacities for high-level activities of daily living and executive functions

The Lawton IADL scale (35) was used to measure the IADLs of the study participants, and the higher-level functional capacities for modern daily living were measured using the JST-IC. The JST-IC was developed based on Lawton's hierarchical model of competence systematized for functional capacities into seven conceptual levels (36), which measures the capacities for higher-level functional activities of modern daily living compared to the conventional IADL scales (19). The JST-IC consists of 16 items in four factors (social engagement, technology usage, information practice, and life management) considering modern lifestyle. Using a dichotomous rating scale (0 = no, 1 = yes; maximum score 16 points), survivors reported whether they could independently perform the activity described in each item. To evaluate executive functions, the six items of the Behavioral Assessment of the Dysexecutive Syndrome (BADS) (37) were used: the Rule Shift Cards Test, Action Program Test, Key Search Test, Temporal Judgment Test, Zoo Map Test, and Modified Six Elements Test. These assessments were also conducted 1 year later, i.e., all data were collected initially and 1 year later.

### Statistical analyses

This study involved two types of analyses: analyses of the difference in each score between the initial test and the same test 1 year later, and analyses of the relationship between the changes in capacities for higher-level activities of daily living and executive functions. Wilcoxon's rank-sum sign test was performed to reveal the difference in each variable between the initial and 1-year assessments. These analyses were based on the results of the Shapiro–Wilk test carried out to identify the changes in the Lawton IADL total scores, the JST-IC (total scores and score for each category), and the BADS (age-adjusted total scores and scores for each question) over 1 year. To evaluate changes in the Lawton IADL total score in relation to changes in the BADS total score, we counted the participants who had a change (improvement or decline) in the Lawton IADL total score and BADS total score, as well as those who had no change. The match percentages were then calculated. Similarly, the participants who had a change or no change in JST-IC were counted, and the match percentages were calculated. Two Spearman's correlation coefficients were calculated to statistically clarify associations between the two measures of the capacities for activities of daily living and executive functions (i.e., the Lawton IADL total score and BADS total score, and the JST-IC total score and BADS total score). All statistical analyses were conducted using SPSS version 25 statistical software (IBM SPSS Statistics for Windows; IBM, Armonk, NY, USA). The level of significance was set at *p* < 0.05.

## Results

**Figure 1:**
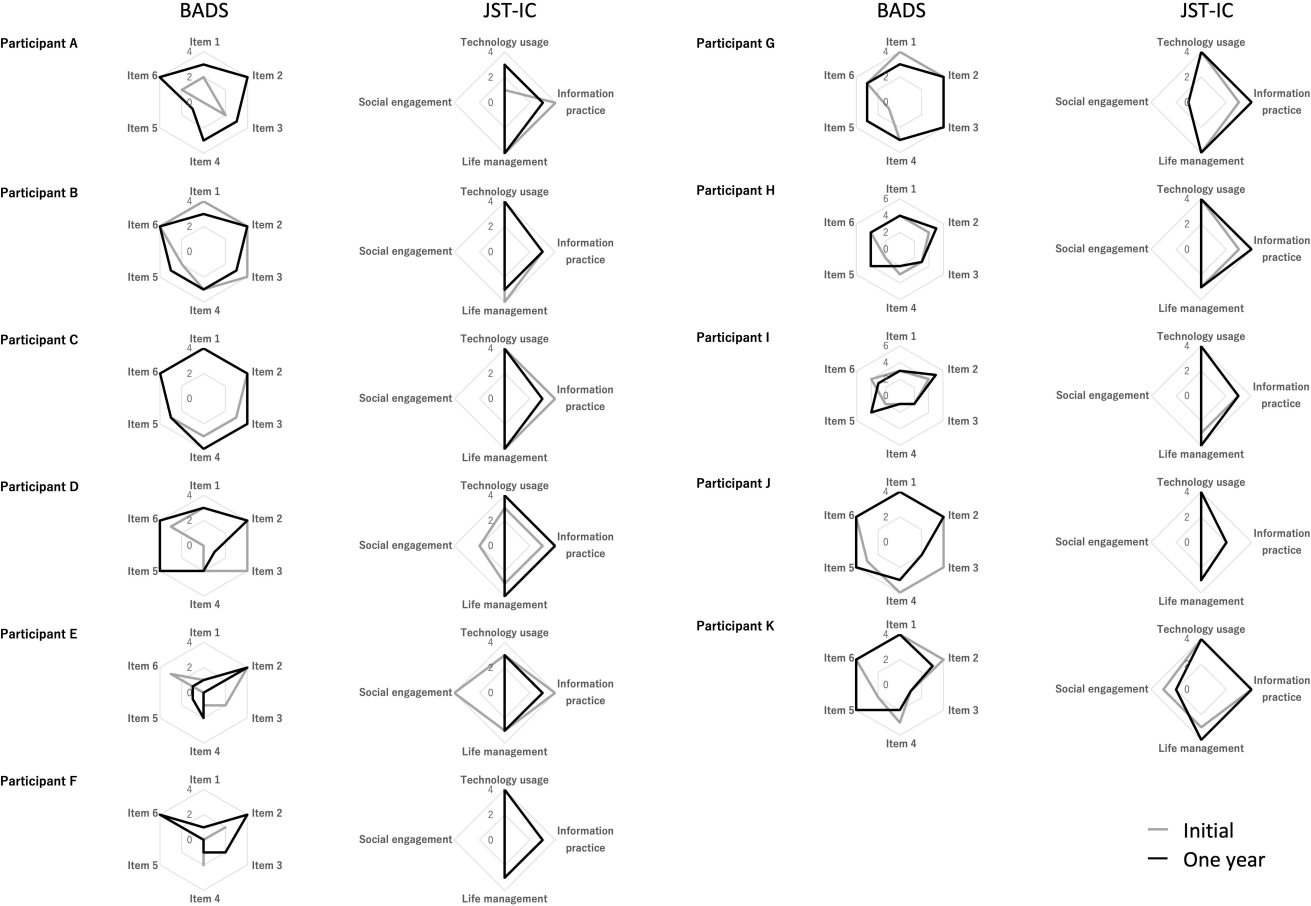
Radar charts of BADS and JST-IC scores at the initial assessment and 1 year later.

**Figure 2:**
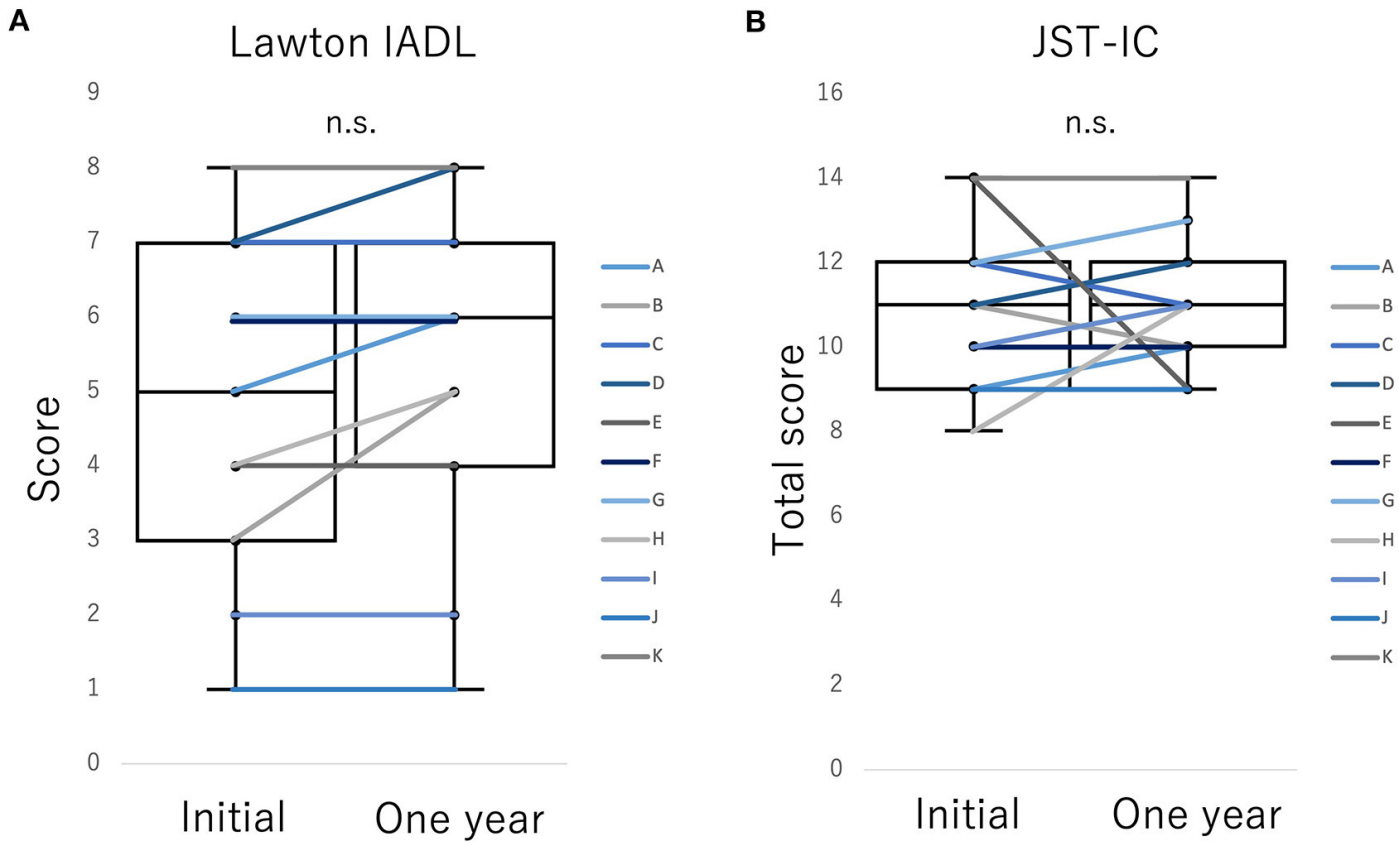
Changes in higher-level functional capacities for daily living **(A)** Lawton IADL, **(B)** JST-IC between initial and 1-year assessments in Participants A–K. The scores for higher capacities for daily living did not change significantly in either of the assessments.

Figure 1 shows the initial and 1-year BADS and JST-IC scores. There were no significant changes in Lawton IADL total scores or in the JST-IC (total scores and scores in each category) between the initial and 1-year assessments (Figure 2). Also, no significant changes in BADS total scores were found. On the other hand, the BADS item 5 score showed a significant increase (*p* = 0.08, Z value = – 2.67), whereas no other significant changes were observed.

In addition, the correspondence between the numbers of participants with improvement, no change, and decline in Lawton IADL score, JST-IC total score, and BADS total score was analyzed. The results showed that 3 participants improved in both Lawton IADL and BADS and 3 participants remained unchanged in both. Thus, 6/11 (54.5%) participants exhibited either changes or no changes in both Lawton IADL and BADS. In JST-IC and BADS, 5 participants improved in both JST-IC and BADS, 2 participants had no change in both, and 2 participants declined in both. Thus, 9/11 (81.8%) participants exhibited either changes or no changes in both scores.

**Figure 3:**
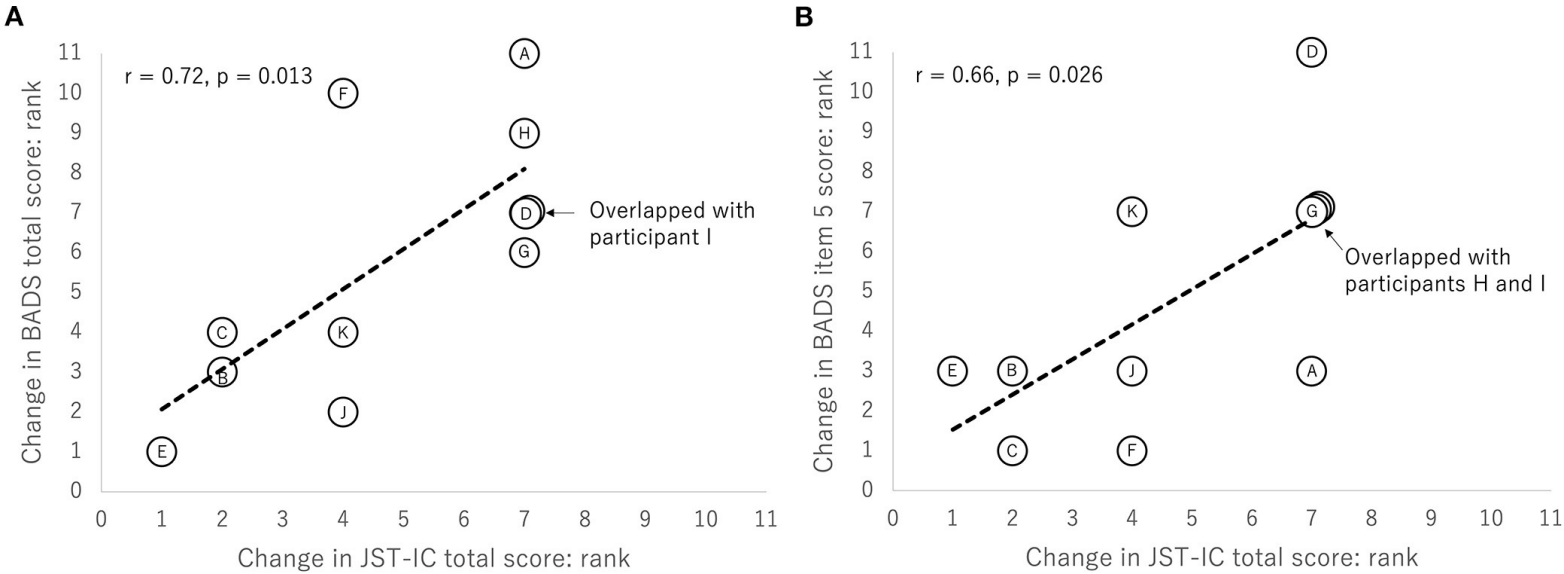
Scatterplots of **(A)** changes in JST-IC total score and in BADS total score and **(B)** changes in JST-IC total score and in BADS item 5 score. All the plots are based on rank. Letters in plots indicate participant IDs.

The JST-IC total score correlated significantly with the BADS total score (*r* = 0.72, *p* = 0.013). Similarly, the JST-IC total score correlated significantly with the BADS item 5 score (*r* = 0.66, *p* = 0.026). Figure 3 shows scatterplots of these relationships. On the other hand, the Lawton IADL score was not significantly correlated with the BADS scores.

## Discussion

In this study, we tested for the first time (a) the 1-year changes in the capacities for higher-level activities of modern daily living and (b) whether executive functions were associated with these capacities in community-dwelling stroke survivors. The JST-IC was used to assess modern lifestyle. The results showed that there were no significant differences in the JST-IC total score or in the Lawton IADL score between the initial and one-year assessments. The variability among participants who improved, declined, or showed no change was confirmed. The consistency rate between participants with either changes or no changes in both the JST-IC and BADS was high (81.8%), and changes in the JST-IC were positively correlated with changes in executive functions as indicated by BADS.

The findings that stroke survivors' capacities for higher-level activities of daily living neither improved nor declined significantly can be interpreted as a positive result. Typically, a decline in basic ADL (38) and IADL abilities (25) in stroke survivors has been strongly associated with aging. Aging has also been reported to be the most important factor in increased frailty in the elderly (39, 40). On the basis of these reports, it is assumed that the capacities for the higher-level activities of daily living generally decline with aging. However, the present results showed no statistically significant decline in higher capacities by the Lawton IADL or JST-IC after 1 year. These results suggest that the performance of daily activities in conjunction with rehabilitation intervention may prevent a decline in the capacities for higher-level activities of daily living. However, we are unable to reach any definitive conclusion about the effects of the rehabilitation intervention because there was no control group in this study. Importantly, however, we were able to measure the capacities for the activities of modern daily living in stroke survivors for the first time. In addition, the results showed that daily living while receiving rehabilitation intervention can maintain the higher capacities. That is, the results are likely to provide essential insights into optimal rehabilitation settings.

There were no changes in BADS total score or in most subscores 1 year later. Only item 5 showed significant improvement. Item 5, the Zoo Map Test, measures performance related to rule-based planning (41). In this test, participants were required to use a map of a zoo to plan a route to visit certain animals. The improvement in item 5 suggests that the maintenance of the capacity for higher-level activities of daily living may have occurred via an improvement in the participants' planning ability.

After 1 year, participants who showed improved higher-level functional capacities for daily living also showed improved BADS scores (and vice-versa). This congruence was particularly strong in the JST-IC (the congruence percentage between BADS and Lawton IADL and that between BADS and JST-IC were 54.5 and 81.8%, respectively). This suggests a linkage between the two. One result supporting such a linkage is the significant correlation between changes in JST-IC scores and changes in BADS scores. The higher-level activities of modern daily living measured by the JST-IC are more complex and challenging compared to the usual IADLs (18, 19). Thus, modern living requires executive functions to support further planning and efficiency. We propose that this may be the mechanism underlying the association between the JST-IC and BADS.

In particular, changes in total JST-IC scores were significantly correlated with changes in the BADS item 5 score (Zoo Map Test). A previous study has shown that capacities for higher-level activities of daily living require planning ability with rule compliance based on BADS item 5 (42). This previous result supports the present results. Changes or no changes in the capacities for higher-level activities of modern daily living as indicated by the JST-IC may be associated with changes or no changes in planning ability based on rule compliance (BADS item 5; i.e., the Zoo Map Test). Therefore, participants with higher improvements in JST-IC scores would be likely to obtain higher improvements in Zoo Map Test scores.

However, although a previous study found that executive functions were associated with Lawton IADL (25), executive functions were not associated with Lawton IADL in the present study. This discrepancy may be related to the capacities for the basic activities of daily living of participants; it is possible, but not certain, that the higher basic ADL abilities (FIM scores) of participants may have influenced this discrepancy.

This study has several limitations. The first is the small number of participants. Because this report is a case series, the results showing individual progress are the most original findings, while the results of the statistical analysis should be considered with caution. To strengthen the robustness of the present results in future studies, the number of participants needs to be increased. Second, this study was a case series and did not have a control group. Thus, we cannot conclude that the rehabilitation was effective at maintaining higher-level functional capacities for daily living. Since it is believed that capacities for the activities of daily living generally decline, the present results suggested that rehabilitation intervention has beneficial effects on these daily living capacities. In future research, comparison with a control group will be necessary in order to fully elucidate the effects of rehabilitation intervention.

In conclusion, this study showed that capacities for the activities of modern daily living can be maintained in community-dwelling stroke survivors. The results suggest that changes in modern daily living capacities may be associated with changes in executive functions. Rehabilitation interventions may be effective for improving executive functions and may promote maintenance of the higher-level activities of modern daily living.

## Data availability statement

The raw data supporting the conclusions of this article will be made available by the authors, without undue reservation.

## Ethics statement

The studies involving human participants were reviewed and approved by the Institutional Ethics Committee of Yokohama Shin-midori General Hospital. The patients/participants provided their written informed consent to participate in this study.

## Author contributions

TK contributed with study concept and design of the survey, analyses, interpretation of data, and drafting and revising the manuscript. MO contributed with study concept and design of the survey, acquisition of data, and revising the manuscript. RE, KM, and HS contributed with acquisition of data and revising the manuscript. KG contributed interpretation of data and revising the manuscript. All authors reviewed and approved the final manuscript.

## Funding

This work was supported by the Grant-in-Aid for JSPS (Japanese Society for Promotion of Science) fellows under Grant Number 18K17732.

## Conflict of interest

The authors declare that the research was conducted in the absence of any commercial or financial relationships that could be construed as a potential conflict of interest.

## Publisher's note

All claims expressed in this article are solely those of the authors and do not necessarily represent those of their affiliated organizations, or those of the publisher, the editors and the reviewers. Any product that may be evaluated in this article, or claim that may be made by its manufacturer, is not guaranteed or endorsed by the publisher.
